# Synthetic studies towards isomeric pyrazolopyrimidines as potential ATP synthesis inhibitors of *Mycobacterium tuberculosis*. Structural correction of reported *N*-(6-(2-(dimethylamino)ethoxy)-5-fluoropyridin-3-yl)-2-(4-fluorophenyl)-5-(trifluoromethyl)pyrazolo[1,5-*α*]pyrimidin-7-amine

**DOI:** 10.1016/j.tetlet.2021.153611

**Published:** 2022-02-02

**Authors:** Peter J. Choi, Guo-Liang Lu, Hamish S. Sutherland, Anna C. Giddens, Scott G. Franzblau, Christopher B. Cooper, William A. Denny, Brian D. Palmer

**Affiliations:** a Auckland Cancer Society Research Centre, School of Medical Sciences, University of Auckland, Private Bag 92019, Auckland 1142, New Zealand; b Maurice Wilkins Centre, University of Auckland, Private Bag 92019, Auckland 1142, New Zealand; c Institute for Tuberculosis Research, College of Pharmacy, University of Illinois at Chicago, 833 South Wood Street, Chicago, IL 60612, USA; d Global Alliance for TB Drug Development, 40 Wall Street, New York, NY 10005, USA

**Keywords:** Pyrazolopyrimidines, Structure-activity relationships, Synthesis, Tuberculosis, Antimicrobial resistance

## Abstract

During our studies into preparing analogues of pyrazolopyrimidine as ATP synthesis inhibitors of *Mycobacterium tuberculosis*, a regiospecific condensation reaction between ethyl 4,4,4-trifluoroacetoacetate and 3-(4-fluorophenyl)-1*H*-pyrazol-5-amine was observed which was dependent on the specific reaction conditions employed. This work identifies optimized reaction conditions to access either the pyrazolo[3,4-*β*]pyridine or the pyrazolo[1,5-*α*]pyrimidine scaffold. This has led to the structural confirmation of the previously reported pyrazolopyrimidine **17b** which was reported as pyrazolo[1,5-*α*]pyrimidine structure **2** which was corrected to pyrazolo[3,4-*β*]-pyrimidine **19**.

## Graphical abstract

**Figure:**
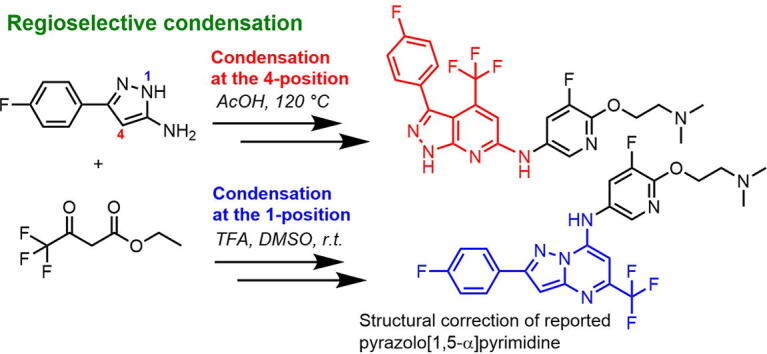

Antimicrobial resistance (AMR) is a global health threat, and the World Health Organisation has declared that AMR is one of the top ten global public health threats facing humanity [1]. AMR occurs when bacteria no longer respond to medicines due to genetic changes, and is often driven by the misuse of antimicrobial agents. Tuberculosis (TB) is one of the leading causes of death from infectious disease globally. In 2019, there were 10 million new cases of active TB, with 1.4 million deaths. Drug resistance against the current standard treatment for *Mycobacterium tuberculosis* (*M.tb*), the causative agent of TB, is on the rise with increases in rifampicin-resistant TB (RR-TB) and multi-drug resistant TB (MDR-TB) being detected. In 2019, treatment success rates of 85% were observed for drug-susceptible TB, while this rate dropped to 56% for MDR-TB [2]. These resistant strains of *M.tb* require longer treatment courses (including injectables), which are more expensive and more difficult to access. The development of novel TB drugs to treat these resistant strains of *M.tb* is urgently needed.

There has been steady progress in the past fifteen years to develop novel drugs for treating TB, with many of these agents currently in clinical trials as part of combination therapy regimens [3]. Novel ATP synthase inhibitors include bedaquiline, which was first drug to be conditionally approved by the FDA in 2012, and its second generation analogues TBAJ-587 and TBAJ-876, which have recently entered phase I clinical trial [4], [5], [6], [7], [8], [9], [10]. There have been recent reports of squaramides [11], tetrahydroisoquinolines [12], 2,4-diaminoquinolines and pyrazolo[1,5-*α*]pyrimidines [13], [14] as potential ATP synthesis pathway inhibitors of *M.tb*. In particular, the pyrazolo[1,5-*α*]pyrimidine scaffold has attracted interest from our research group, with previous compounds having shown potent activity against *M.tb* [13], [14]. Derivatives with this scaffold have also been shown to be potential antianxiety agents [15] and the structurally similar triazolopyrimidines were reported to have antimalarial [16], [17], [18] and antileishmaniasis [19] properties.

Recent work from Tantry and co-workers has described pyrazolo[1,5-*α*]pyrimidines as potent ATP synthesis pathway inhibitors for the treatment of tuberculosis [13]. Our group has also been focused on elucidating structural activity relationships of novel pyrazolo[1,5-*α*]pyrimidines as potential inhibitors of *M.tb*. During the initial screening of a series of pyrazolo[1,5-*α*]pyrimidines for inhibition of ATP synthesis, we attempted to resynthesize compounds **1** and **2** (compound **17a** and **17b**, respectively, from Tantry and co-workers) as starting points for our drug discovery campaign (Fig. 1).

**Fig. 1:**
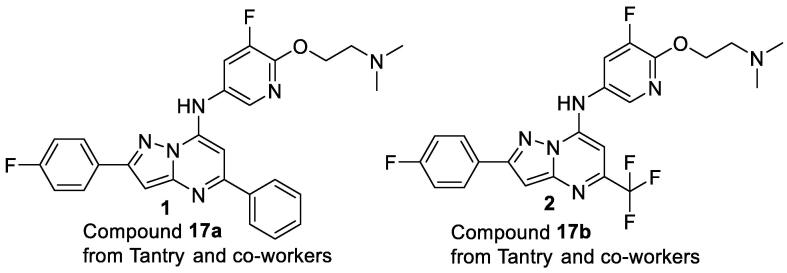
Pyrazolo[1,5-*α*]pyrimidines synthesized by Tantry and co-workers as potent ATP synthesis pathway inhibitors for the treatment of tuberculosis.

Synthesis of **1** was achieved following the reported reaction conditions (Scheme 1). Condensation of 3-(4-fluorophenyl)-3-oxopropanenitrile **3** with hydrazine hydrate gave 3-(4-fluorophenyl)-1*H*-pyrazol-5-amine **4**. Reaction of aminopyrazole **4** with β-keto ester **5** gave pyrazolopyrimidinone intermediate **6** exclusively. Treatment of pyrazolopyrimidinone **6** with phosphorous oxychloride provided **7**, which underwent nucleophilic substitution with 6-[2-(dimethylamino)ethoxy]-5-fluoropyridin-3-amine **8** to give **1** in poor yield. This reaction could be improved using Buchwald-Hartwig amination reaction conditions to afford compound **1**.

**Scheme 1:**
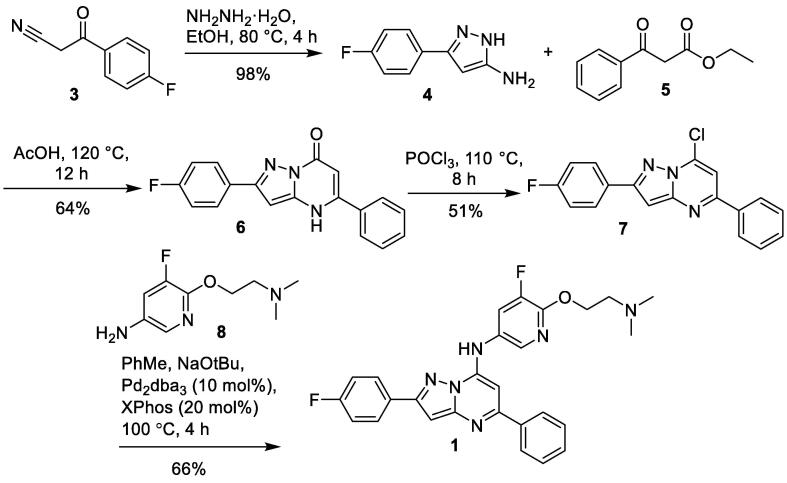
Synthesis of pyrazolo[1,5-*α*]pyrimidine **1**.

Synthesis of target compound **2** was more challenging. Following the reported condensation reaction conditions [13], reaction of aminopyrazole **4** and ethyl 4,4,4-trifluoroacetate **9** in acetic acid at 120 °C for 4 h gave the alternative cyclized pyrazolo[3,4-*β*]pyridinone **11,** instead of the desired pyrazolo[1,5-*α*]pyrimidinone **10** (Scheme 2).

**Scheme 2:**
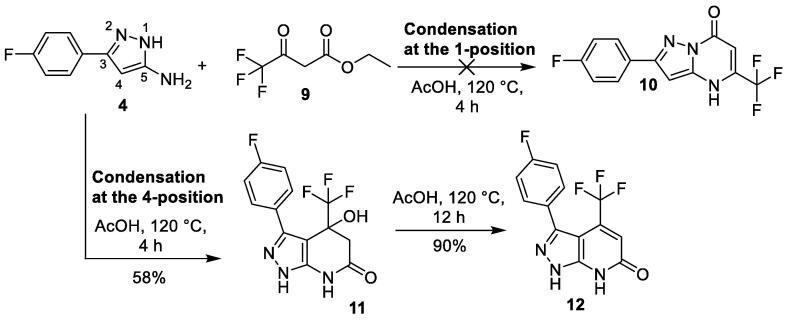
Synthesis of pyrazolo[3,4-*β*]pyridinone **12**.

This could be explained if the condensation reaction had occurred at the 4-position of aminopyrazole **4**, instead of at the usual 1-position which was observed in the previous formation of pyrazolo[1,5-*α*]pyrimidinone **6** (Scheme 1). Aminopyrazoles typically undergo condensation reactions at the 1-position with symmetrical β-diketones or β-keto esters to yield the pyrazolo[1,5-*α*]pyrimidinone scaffold [14]. However in rare cases, this condensation reaction undergoes condensation at the 4-position of aminopyrazoles to yield pyrazolo[3,4-*β*]pyridinones. This was observed when the β-keto ester ethyl 4,4,4-trifluoroacetate **9** was used in the condensation reaction with various aminopyrazoles [20], [21]. The electron withdrawing CF_3_ group in ethyl 4,4,4-trifluoroacetate **9**, makes it sufficiently activated for electrophilic attack at the reactive 4-position of the aminopyrazole ring to take place.

With the condensation reaction forming the undesired pyrazolo[3,4-*β*]pyridinone core **11**, alternative methods to access pyrazolo[1,5-*α*]pyrimidinone core **10** were investigated. There have been reports of similar reactions between ethyl 4,4,4-trifluoroacetate **9** and aminopyrazoles where the 4-position of the pyrazole ring was blocked with a methyl group to prevent the condensation reaction occurring at this position. This led to exclusive formation of pyrazolo[1,5-*α*]pyrimidinones [22]. However this method was not feasible as our aminopyrazole **4** is required to be unsubstituted at the 4-position.

Similar reactions between the related aminopyrazole **13** and hexafluoroacetylacetone **14** have been reported in the literature (Scheme 3). This reaction was reported first by Nam and co-workers which outlines exclusive formation of pyrazolo[3,4-*β*]pyridine **16** when the reaction was heated to 140 °C without solvent (condition 1) [23]. However, Petrov and co-workers report the same reaction could yield the other regioisomer pyrazolo[1,5-*α*]-pyrimidine **15** by altering the reaction conditions [24]. They found that carrying out the reaction at lower temperatures (20 °C) in DMSO gave exclusive formation of pyrazolo[1,5-*α*]-pyrimidine **15** (condition 2).

**Scheme 3:**
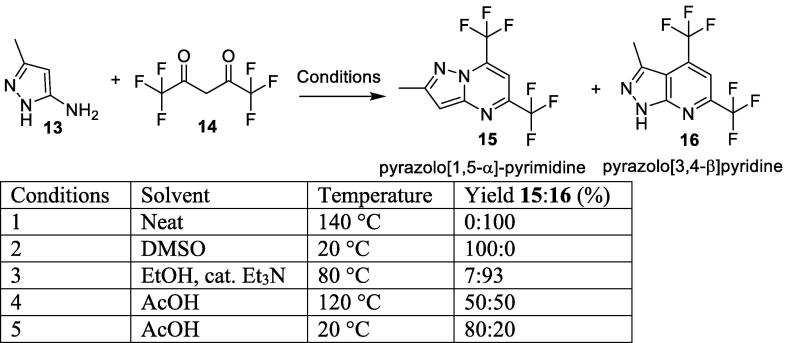
Formation of pyrazolo[3,4-*β*]pyridine **16** and pyrazolo[1,5-*α*]-pyrimidine **15** scaffold.

Encouraged by these results, aminopyrazole **4** and ethyl 4,4,4-trifluoroacetate **9** were dissolved in DMSO and stirred at r.t for 24 h (Scheme 4). However, no reaction occurred with both starting materials still present after 48 h. The reaction was repeated with catalytic amounts of trifluoroacetic acid as an acid catalyst. After 24 h, desired pyrazolo[1,5-*α*]-pyrimidinone **10** was isolated in 59% yield as the sole product. Trifluoroacetic acid was the catalyst of choice, as acetic acid readily reacted with the NH_2_ group of aminopyrazole **4** to form the unwanted acetamide byproduct.

**Scheme 4:**
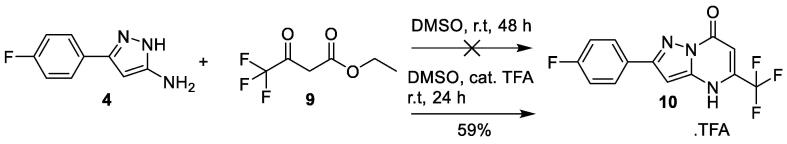
Formation of pyrazolo[1,5-*α*]pyrimidinone **10**.

The structure of **10** was confirmed unambiguously by single-crystal X-ray crystallography (Fig. 2) [25]. A single crystal of **10** was obtained by slow recrystallization by diffusing *n*-hexanes into a dichloromethane solution of **10**. The X-ray crystal structure confirms the desired pyrazolo[1,5-*α*]-pyrimidinone core.

**Fig. 2:**
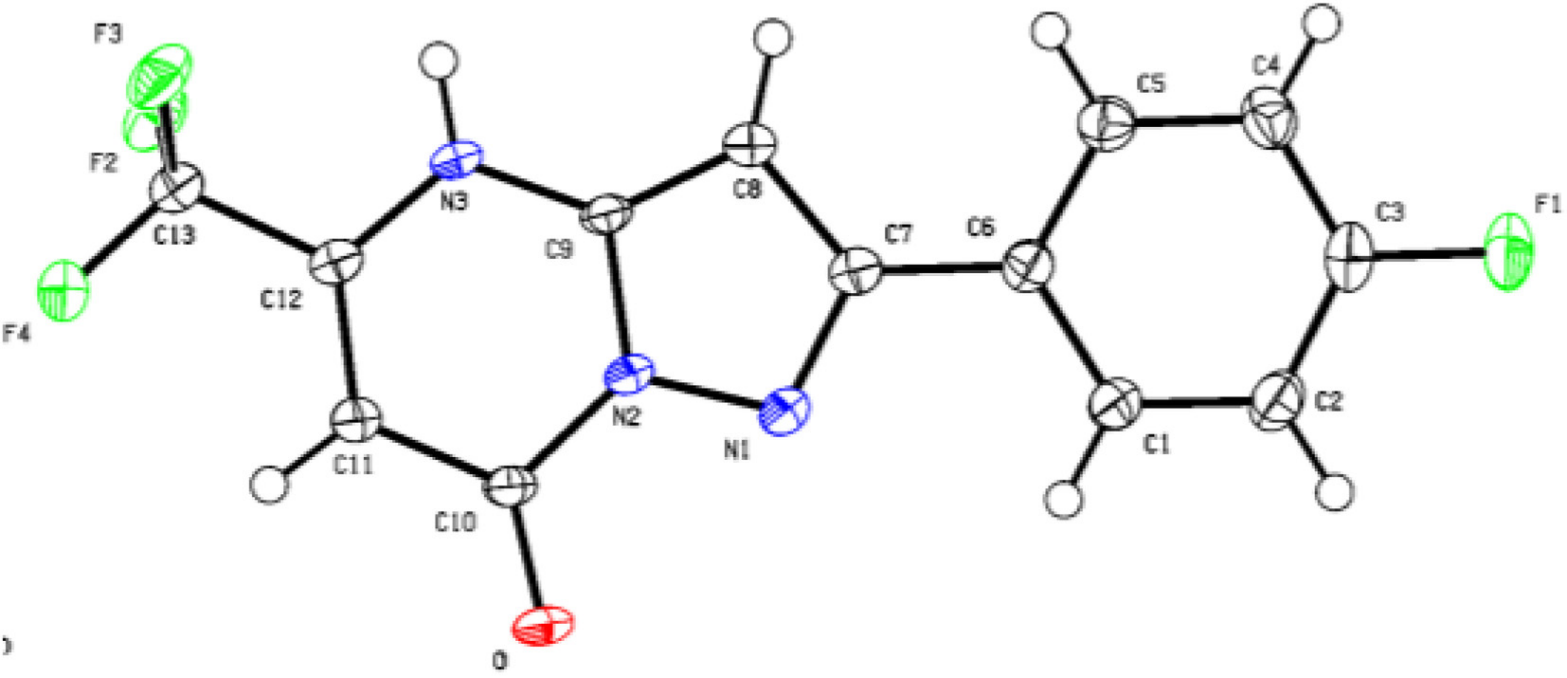
X-ray crystal structure of **10**. Data for crystal structure **10** is available from CCDC 2105641.

Pyrazolo[3,4-*β*]-pyrimidinone **11** and pyrazolo[1,5-*α*]-pyrimidinone **10** were converted to the corresponding chlorides **17** and **18** respectively, using phosphorous oxychloride (Scheme 5). Buchwald-Hartwig amination with **8** gave the final products pyrazolo[3,4-*β*]-pyrimidine **19** and pyrazolo[1,5-*α*]-pyrimidine **2** respectively.

**Scheme 5:**
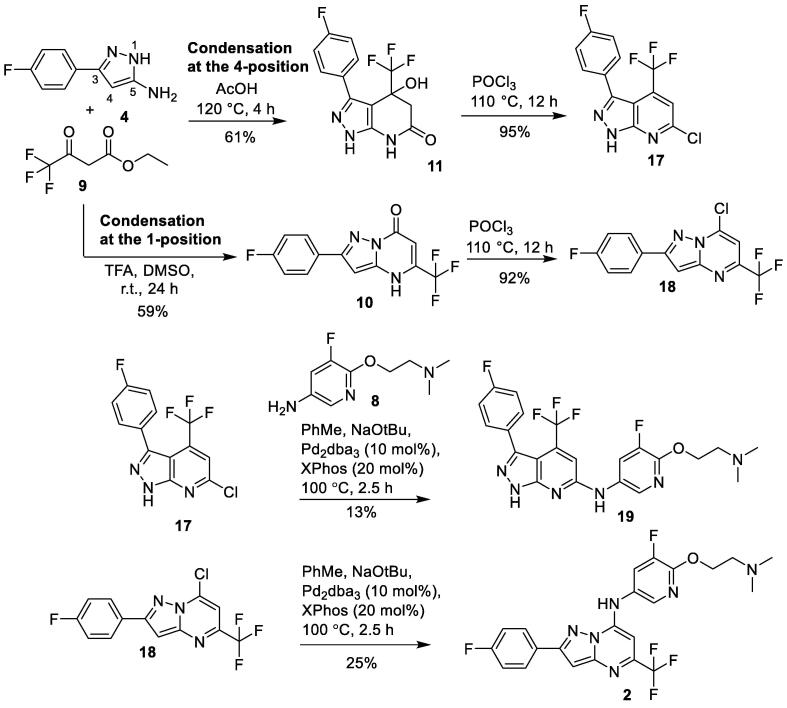
Synthesis of pyrazolo[3,4-*β*]-pyrimidine **19** and pyrazolo[1,5-*α*]-pyrimidine **2**.

From the analytical data generated through this work, it was now clear that the structure of compound **17b** reported by Tantry and co-workers [13] is actually pyrazolo[3,4-*β*]-pyrimidine **19**. The ^1^H NMR spectra of **17b** and **19** matched, except a NH peak at 13.7 ppm was missing in **17b** and it also had an extra peak at 6.68 ppm (Table 1). For ^1^H NMR spectrum of **2** and **19**, please refer to the ESI) [26]. This could be explained if the peak at 6.68 ppm is a ‘folded in’ or ‘aliased’ peak of the true peak at 13.7 ppm. This can be caused by the narrow spectral width setting which was insufficient to encompass all the peaks in the spectrum [27]. The ^1^H NMR spectrum of **19** showed it clearly had two NH’s present (confirmed by D_2_O exchange), while the ^1^H NMR spectrum of pyrazolo[1,5-*α*]-pyrimidine **2** clearly indicates it contains only one NH.

**Table 1:** ^1^H NMR comparison of reported **17b** and pyrazolo[3,4-*β*]-pyrimidine **19** in DMSO, 400 MHz.

Position	**17b**^1^H NMR δ ^a^	**19**^1^H NMR δ
1-NH	–	13.7 (s, 1H)
NH	10.3 (s, 1H)	10.0 (s, 1H)
2′, 6′	8.30–8.34 (m, 2H)	8.31–8.34 (m, 2H)
3′, 5′	7.45–7.48 (m, 2H)	7.46–7.50 (m, 2H)
5, 4″	7.25–7.29 (m, 2H)	7.26–7.31 (m, 2H)
2″	7.05 (s, 1H)	7.06 (s, 1H)
‘Aliased’ 1-NH	6.68 (s, 1H)	–
O-CH_2_	4.42–4.44 (m, 2H)	4.42 (t, *J* = 5.9 Hz, 2H)
N-CH_2_	2.76 (m, 2H)	2.66 (t, *J* = 5.8 Hz, 2H)
N(CH_3_)_2_	2.29 (s, 6H)	2.23 (s, 6H)

^a^ Data published by Tantry and co-workers [13].

Minimum inhibitory concentration values for pyrazolo[1,5-*α*]-pyrimidines **2** and pyrazolo[3,4-*β*]-pyrimidine **19** were determined against both aerobic (replicating; MABA) and anaerobic (non-replicating; LORA) cultures of *M.tb* (Table 2). Results indicate both analogues possess moderate MIC_90_ values of ∼8 µg/mL against MABA bacterial cultures and ∼11 µg/mL against LORA cultures. The compounds were additionally tested for mammalian cell toxicity (with Vero green monkey-derived epithelial kidney cells), where both compounds had IC_50_s 11 ∼ 12 μg/mL. In comparison, the value for bedaquiline in repeat assays was between 4 and 16 μg/mL.

**Table 2:** Inhibitory properties of pyrazolo[1,5-*α*]-pyrimidines **2** and pyrazolo[3,4-*β*]-pyrimidine **19**.

Compound	MABA MIC_90_^a^ (µg/mL)	LORA MIC_90_^a^ (µg/mL)	VERO IC_50_^b^(µg/mL)
**Bedaquiline^c^**	0.04	0.08	4 ∼ 16 in repeat assays
**2**	7.9	10.9	12.0
**19**	7.70	10.95	11.43

^a^MIC_90_ (µg/mL); minimum inhibitory concentration for 90% inhibition of growth of *M.tb* strain H37Rv, determined under aerobic (replicating; MABA) [28] or non-replicating (LORA) [29] conditions, determined at the Institute for Tuberculosis Research, University of Illinois at Chicago. Each value is the mean of at least two independent determinations; ^b^IC_50_ values (µg/mL) in green monkey kidney epithelial (VERO) cells as a measure of mammalian cell toxicity [30]; ^c^Bedquiline is a known ATP synthase inhibitor for treatment of multi-drug-resistant tuberculosis.

A unique condensation reaction between ethyl 4,4,4-trifluoroacetate **9** and 3-(4-fluorophenyl)-1*H*-pyrazol-5-amine **4** has resulted in two distinct regioisomers depending on the reaction conditions. Condensation reaction conditions using ethanol with acetic acid at high temperatures resulted in the formation of pyrazolo[3,4-*β*]-pyrimidinone **11**, while the same reaction carried out in DMSO at room temperature with catalytic TFA resulted in the exclusive formation of the pyrazolo[1,5-*α*]-pyrimidinone **10**. This provides a unique regioselective method for accessing both regioisomers and highlights that care must be taken when assigning structures of products derived from condensation reactions with CF_3_-substituted β-keto esters. This chemistry was successfully utilized in synthesizing the analogues pyrazolo[1,5-*α*]-pyrimidines **2** and pyrazolo[3,4-*β*]-pyrimidine **19** as potential ATP synthesis inhibitors of *M.tb*. The reported structure of **17b** has been confirmed and corrected with X-ray crystallography and ^1^H NMR spectroscopy as pyrazolo[3,4-*β*]-pyrimidine **19**.

## Declaration of Competing Interest

The authors declare that they have no known competing financial interests or personal relationships that could have appeared to influence the work reported in this paper.
