# Supplementary material for: Synthetic studies towards isomeric pyrazolopyrimidines as potential ATP synthesis inhibitors of Mycobacterium tuberculosis. Structural correction of reported N-(6-(2-(dimethylamino)ethoxy)-5-fluoropyridin-3-yl)-2-(4-fluorophenyl)-5-(trifluoromethyl)pyrazolo[1,5-α]pyrimidin-7-amine
Source: Tetrahedron Lett. 2022 Feb 2;90:None. doi: 10.1016/j.tetlet.2021.153611 (PMC8809387; doi:10.1016/j.tetlet.2021.153611)
Supplement: Supplementary data 1 [file mmc1.docx]

**Supplementary Data**

Synthetic studies towards isomeric pyrazolopyrimidines as potential ATP synthesis inhibitors of *Mycobacterium tuberculosis*. Structural correction of reported *N*-(6-(2-(dimethylamino)ethoxy)-5-fluoropyridin-3-yl)-2-(4-fluorophenyl)-5-(trifluoromethyl)pyrazolo[1,5-α]pyrimidin-7-amine.

Peter J. Choi^1,2*^, Guo-Liang Lu^1,2^, Hamish S. Sutherland^1,2^, Anna C. Giddens^1^, Scott G. Franzblau^3^, Christopher B. Cooper^4^, William A. Denny^1,2^ and Brian D. Palmer^1,2^

^1^ Auckland Cancer Society Research Centre, School of Medical Sciences, University of Auckland, Private Bag 92019, Auckland 1142, New Zealand

^2^ Maurice Wilkins Centre, University of Auckland, Private Bag 92019, Auckland 1142, New Zealand

^3^ Institute for Tuberculosis Research, College of Pharmacy, University of Illinois at Chicago, 833 South Wood Street, Chicago, Illinois 60612, USA

^4^ Global Alliance for TB Drug Development, 40 Wall Street, New York, New York 10005, USA

***** *Corresponding author*.

Email address: [p.choi@auckland.ac.nz](mailto:p.choi@auckland.ac.nz)

**General Experimental Methods**.

All reactions were carried out under an atmosphere of dry nitrogen. Glasswares were

oven-dried prior to use. Unless otherwise indicated, common reagents or materials were obtained from commercial sources and used without further purification. All solvents were dried prior to use with appropriate drying agents. Dry distilled DMF, Toluene were obtained from Acros and used as such. Flash column chromatography was performed using silica gel 60 (230-400 mesh). Analytical thin layer chromatography (TLC) was carried out on Merck silica gel plates with QF-254 indicator and visualized by UV. Fluorescence spectra were obtained on a Varian Cary Eclipse fluorescence spectrophotometer at room temperature. Absorbance spectra were obtained on a Varian 100 Bio UV-Vis spectrophotometer at room temperature. ^1^H and ^13^C NMR spectra were obtained with a Bruker Avance 400 spectrometer at 400 MHz for ^1^H and 101 MHz for ^13^C spectra. Spectra were obtained in CDCl_3_ or (CD_3_)_2_SO. The chemical shifts are reported in parts per million (d) downfield using tetramethylsilane (SiMe_4_) as internal standard. Spin multiplicities are given as s (singlet), d (doublet), dd (double doublet), br (broad), m (multiplet), and q (quartet). Coupling constants (J values) were measured in hertz (Hz). All LC/MS data were gathered by direct injection of methanolic solutions into a Surveyor MSQ mass spectrometer using an atmospheric pressure chemical ionisation (APCI) with a corona voltage of 50 V and a source temperature of 400 °C. High-resolution electrospray ionisation (HRESIMS) mass spectra were determined on a Bruker micrOTOFQ II mass spectrometer.

**2-(4-Fluorophenyl)-5-phenylpyrazolo[1,5-α]pyrimidin-7(4*H*)-one 6**

A mixture of 3-(4-fluorophenyl)-1*H*-pyrazol-5-amine (3.15 g, 17.78 mmol) and ethyl 3-oxo-3-phenylpropanoate (3.37 g, 19.56 mmol) in acetic acid (30 mL) was refluxed for 12 h. The resulting white suspension was cooled down to room temperature, filtered and washed with diethyl ether to give **6** as a white solid (3.50 g, 64%). ^1^H NMR (*d^6^*-DMSO, 400 MHz) δ 12.61 (s, 1H), 8.04-8.09 (m, 2H), 7.84-7.88 (m, 2H), 7.57-7.62 (m, 3H), 7.29-7.35 (m, 2H), 6.66 (s, 1H), 6.10 (s, 1H). APCI MS m/z [M + H]+: 306.2. *Matches literature MS and ^1^H NMR data.^1^


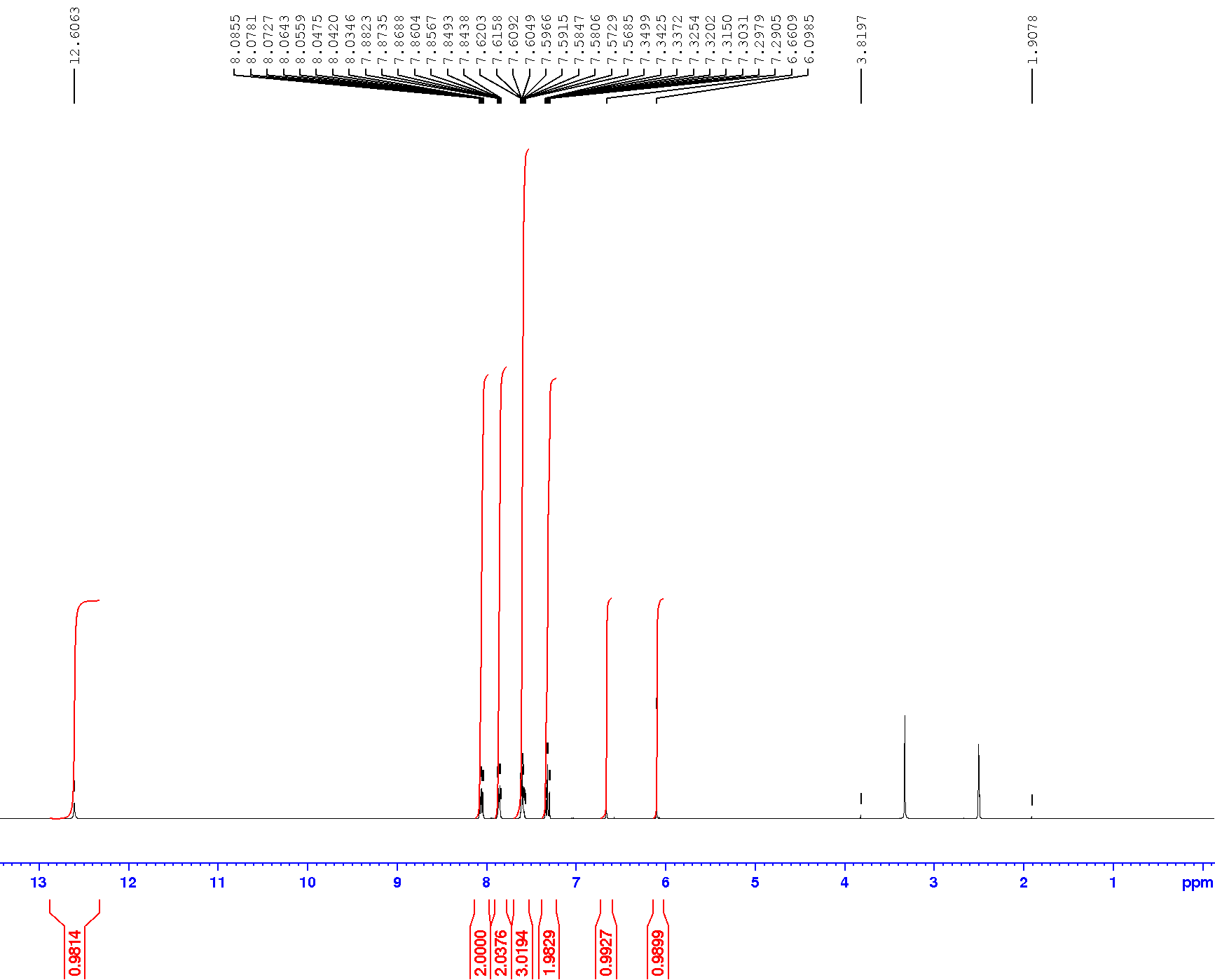


**Figure 1.** ^1^H NMR spectrum of **6**

**7-Chloro-2-(4-fluorophenyl)-5-phenylpyrazolo[1,5-α]pyrimidine 7**

2-(4-Fluorophenyl)-5-phenylpyrazolo[1,5-a]pyrimidin-7(4*H*)-one **6** (3.30 g, 11.10 mmol) was added phosphorous oxychloride (34 mL) and the reaction was heated at 110 °C in a sealed tube for 8 h. Excess phosphorous oxychloride was removed by in vacuo and the mixture was partitioned between EtOAc and water, followed by washing with sat. NaHCO_3_ solution. The organic fractions were collected, dried over anhydrous Na_2_SO_4_ and filtered. After the solvent was removed under reduced pressure, the crude product obtained was further purified by column chromatography to give to give **7** (1.82 g, 51%) as a colourless solid. ^1^H NMR (CDCl_3_, 400 MHz) δ 8.07-8.09 (m, 2H), 8.01-8.05 (m, 2H), 7.52-7.56 (m, 3H), 7.41 (s, 1H), 7.15-7.20 (m, 2H), 7.04 (s, 1H). ^13^C NMR (CDCl_3_, 101 MHz) δ 165.0, 162.6, 156.5, 156.2, 151.2, 138.9, 136.7, 131.0, 129.3, 128.9, 128.9, 128.8, 127.5, 116.2, 116.0, 105.8, 95.3. APCI MS m/z [M + H]+: 324.1. HRMS (m/z): calculated for C_18_H_11_ClFN_3_: 323.0626: found: 323.0636. *Matches literature MS^2^


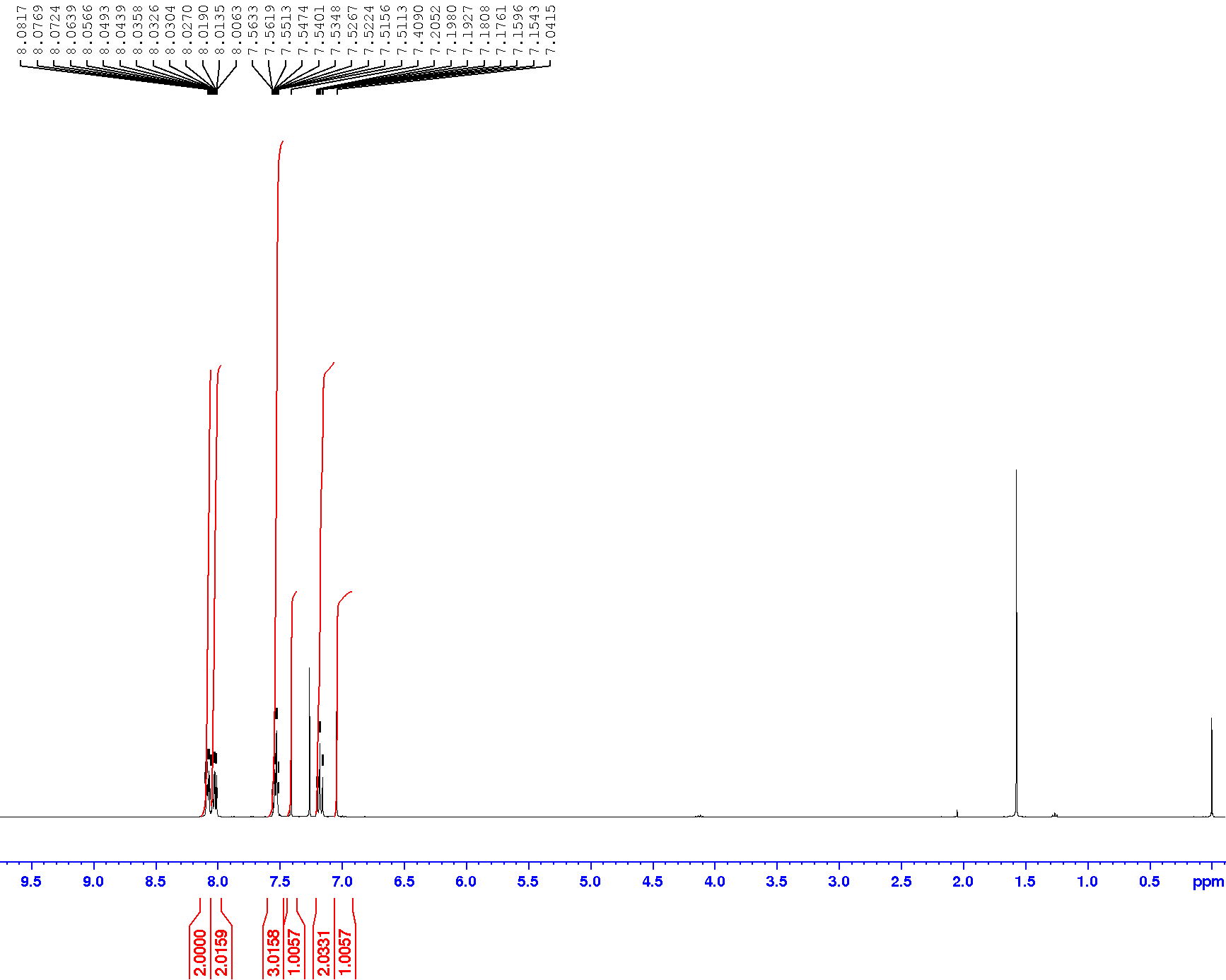


**Figure 2.** ^1^H NMR spectrum of **7**

**Figure 3.** ^13^C NMR spectrum of **7**

**N-(6-(2-(Dimethylamino)ethoxy)-5-fluoropyridin-3-yl)-2-(4-fluorophenyl)-5-phenylpyrazolo[1,5-α]pyrimidin-7-amine 1**

Chloride **7** (0.300 g, 0.93 mmol) in toluene (10 mL) was added 6-(2-(dimethylamino)ethoxy)-5-fluoropyridin-3-amine (0.203 g, 1.02 mmol) and flushed with nitrogen for 5 min. XPhos (0.022 g, 0.05 mmol) and Pd_2_dba_3_ (0.021 g, 0.02 mmol) were added to the reaction mixture and heated to 100 °C for 30 min. After 30 min, NaOtBu (0.134 g, 1.39 mmol) was added and the reaction was heated at 100 °C for further 4 h. Reaction mixture was concentrated under reduced pressure and purified by column chromatography (Al_2_O_3_, standard 90) using gradient mixtures of EtOAc and petrol ether (v/v 1:6 and 1:3) as eluent to give the product **1** as a slightly yellow solid (0.296 g, 66%). HPLC purity 98.8%. ^1^H NMR (CDCl_3_, 400 MHz) δ 8.10 (d, *J* = 2.3 Hz, 1H), 7.99-8.03 (m, 2H), 7.94-7.97 (m, 3H), 7.44-7.51 (m, 4H), 7.16-7.20 (m, 2H), 6.88 (s, 1H), 6.51 (s, 1H), 4.57 (t, *J* = 5.7 Hz, 2H), 2.81 (t, *J* = 5.7 Hz, 2H), 2.38 (s, 6H). ^13^C NMR (CDCl_3_, 101 MHz) δ 164.8, 162.4, 158.2, 155.2, 152.3, 150.5, 148.6, 146.0, 145.5, 138.6, 138.3, 138.2, 130.2, 129.4, 129.3, 129.0, 128.6, 128.5, 127.5, 126.9, 126.9, 121.4, 121.3, 116.1, 115.9, 93.5, 84.1, 65.2, 58.2, 46.2. APCI MS m/z [M + H]+: 487.2. HRMS (m/z): calculated for C_27_H_24_F_2_N_6_O: 486.1980: found: 486.1985. *Matches literature MS and ^1^H NMR data.^2^

**Figure 4.** ^1^H NMR spectrum of **1**

**Figure 5.** ^13^C NMR spectrum of **1**

**3-(4-Fluorophenyl)-4-hydroxy-4-(trifluoromethyl)-1,4,5,7-tetrahydro-6*H*-pyrazolo[3,4-β]pyridin-6-one 11**

3-(4-Fluorophenyl)-1*H*-pyrazol-5-amine (1.58 g, 8.92 mmol) was added ethyl 4,4,4-trifluoro-3-oxobutanoate (1.87 g, 10.7 mmol) and acetic acid (1.5 mL). The reaction was heated at 100 °C in a sealed tube for 4 h. The reaction was cooled to r.t. and solid was filtered and dried to give **11** (1.71 g, 61%) as a colourless solid which was used crude for the next step. ^1^H NMR (DMSO, 400 MHz) δ 12.79 (s, 1H), 10.7 (s, 1H), 7.89 (ddd, J = 11.9, 5.2, 3.0 Hz, 2H), 7.29 (td, J = 8.9, 2.2 Hz, 2H), 7.02 (s, 1H), 2.98 (d, J = 16.5 Hz, 1H), 2.78 (d, J = 16.4 Hz, 1H). ^13^C NMR (DMSO-d6, 101 MHz) δ 167.2, 163.4, 161.0, 149.6, 140.3, 130.7, 130.6, 130.6, 128.5, 128.4, 127.5, 127.5, 127.3, 125.6, 124.4, 115.8, 115.7, 115.6, 115.4, 115.2, 115.0, 97.9, 87.1, 70.9, 70.6, 70.3, 70.0, 41.7. APCI MS m/z [M + H]+: 316.2. HRMS (m/z): calculated for C_13_H_9_F_4_N_3_O_2_: 315.0631: found: 315.0620.^3^


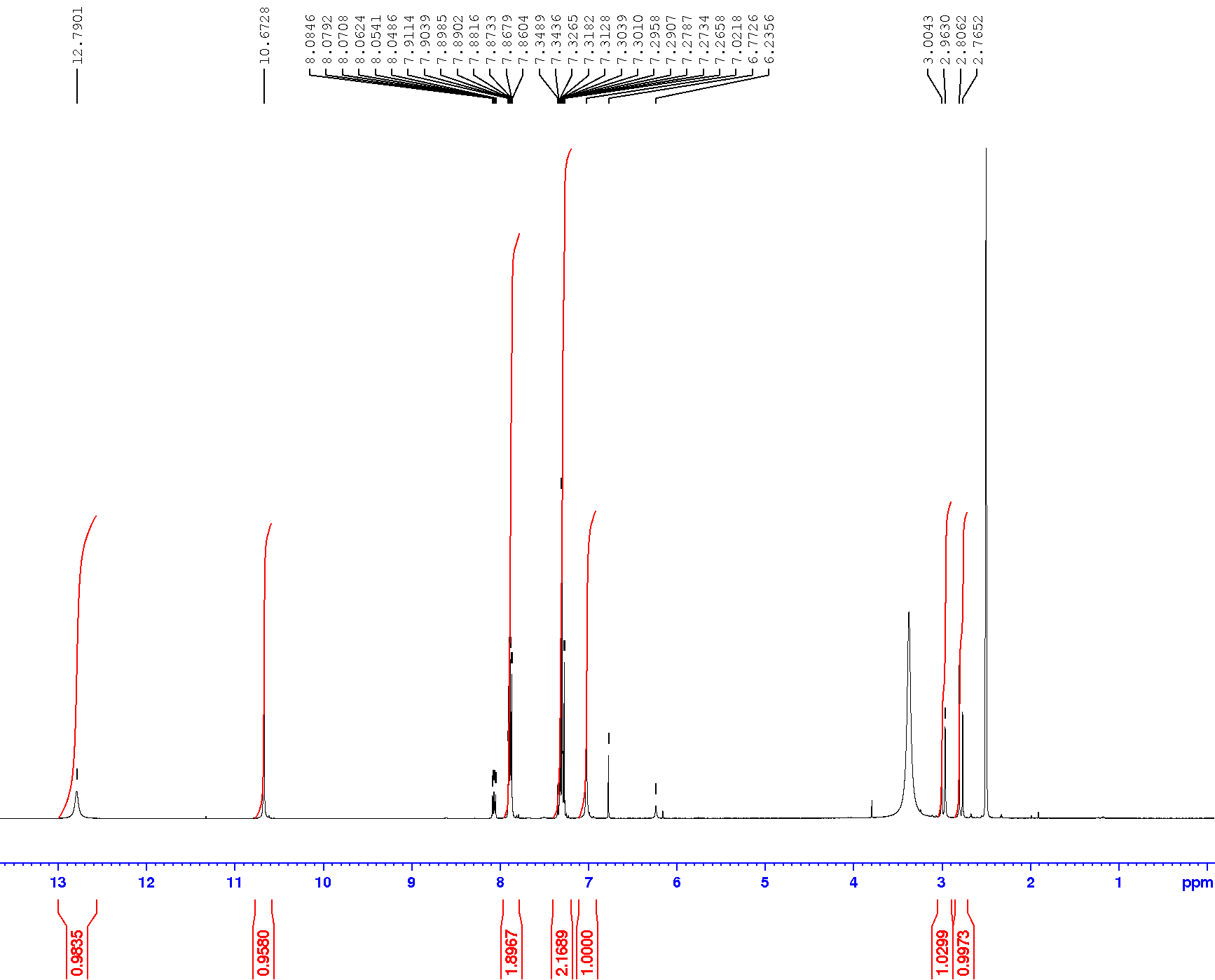


**Figure 6.** ^1^H NMR spectrum of **11**

**Figure 7.** ^13^C NMR spectrum of **11**

**6-Chloro-3-(4-fluorophenyl)-4-(trifluoromethyl)-1*H*-pyrazolo[3,4-β]pyridine 17**

3-(4-Fluorophenyl)-4-hydroxy-4-(trifluoromethyl)-1,4,5,7-tetrahydro-6*H*-pyrazolo[3,4-β]pyridin-6-one **11** (0.366 g, 1.23 mmol) was added phosphorous oxychloride (3 mL) and the reaction was heated at 110 °C in a sealed tube for 12 h. Excess phosphorous oxychloride was removed by in vacuo and the mixture was partitioned between EtOAc and water, followed by washing with sat. NaHCO_3_ solution. The organic fractions were collected, dried and evaporated to give to give **17** (0.37 g, 95%) as a colourless solid which was used crude for the next step. APCI MS m/z [M + H]+: 316.1. HRMS (m/z): calculated for C_13_H_6_ClF_4_N_3_: 315.0186: found: 315.0172. *Matches literature MS.^2^

***N*-(6-(2-(Dimethylamino)ethoxy)-5-fluoropyridin-3-yl)-3-(4-fluorophenyl)-4-(trifluoromethyl)-1*H*-pyrazolo[3,4-β]pyridin-6-amine 19**

Chloride **17** (1.00 g, 3.17 mmol) in toluene (15 mL) was added 6-(2-(dimethylamino)ethoxy)-5-fluoropyridin-3-amine (0.651 g, 3.26 mmol) and flushed with nitrogen for 5 min. XPhos (0.302 g, 0.634 mmol) and Pd_2_dba_3_ (0.291 g, 0.317 mmol) were added to the reaction mixture and heated to 100 °C for 30 min. After 30 min, NaOtBu (0.609 g, 6.34 mmol) was added and the reaction was heated at 100 °C for further 1 h. Reaction mixture was concentrated under reduced pressure and purified by flash column chromatography with silica using EtOAc:MeOH (95:5) to give **19** (0.204 g, 13%). HPLC purity 96.0%. MP=204-207 °C. ^1^H NMR (DMSO, 400 MHz) δ 13.70 (s, 1H), 10.00 (s, 1H), 8.32 (td, J = 6.6. 2.2 Hz, 2H), 7.47 (dd, J = 8.5, 5.7 Hz, 2H), 7.31-7.26 (m, 2H), 7.06 (s, 1H), 4.42 (t, J = 5.9 Hz, 2H), 2.66 (t, J = 5.8 Hz, 2H), 2.23 (s, 6H). ^13^C NMR (DMSO-d6, 101 MHz) δ 163.3, 160.9, 154.2, 152.8, 147.2, 147.1, 146.9, 144.4, 142.8, 131.9, 131.9, 131.6, 131.5, 131.5, 130.9, 130.6, 130.4, 126.5, 123.8, 121.0, 116.2, 116.0, 114.8, 114.6, 105.8, 100.8, 63.8, 57.5, 45.4. APCI MS m/z [M + H]+: 479.2. HRMS (m/z): calculated for C_22_H_19_F_5_N_6_O: 478.1540: found: 478.1536. *Matches literature MS and ^1^H NMR data.^2^


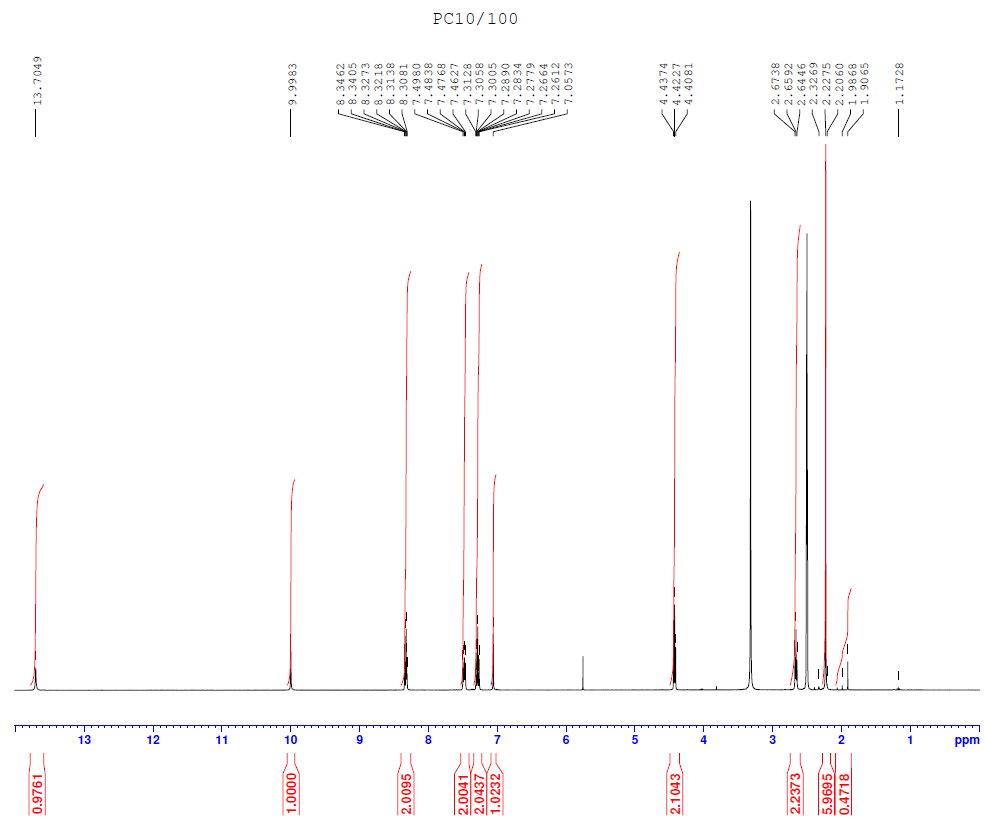


**Figure 8.** ^1^H NMR spectrum of **19**

**Figure 9.** ^13^C NMR spectrum of **19**

**2-(4-Fluorophenyl)-5-(trifluoromethyl)pyrazolo[1,5-α]pyrimidin-7(4*H*)-one 10**

3-(4-Fluorophenyl)-1*H*-pyrazol-5-amine (1.00 g, 5.64 mmol) in DMSO (10 mL) was added ethyl 4,4,4-trifluoro-3-oxobutanoate (1.14 g, 6.20 mmol) and TFA (0.2 mL). The reaction stirred at r.t. for 24 h. The solid was filtered and dried to give **10** (0.99 g, 59%) as a colourless solid which was used crude for the next step. ^1^H NMR (DMSO, 400 MHz) δ 8.06 (ddd, J = 12.2, 5.5, 3.3 Hz, 2H), 7.32 (td, J = 8.9, 2.1 Hz, 2H), 6.77 (s, 1H), 6.25 (s, 1H), 3.86 (br s, 2H). ^13^C NMR (DMSO-d6, 101 MHz) δ 164.0, 161.6, 155.2, 152.9, 128.6, 128.6, 128.3, 128.3, 121.5, 118.8, 115.9, 115.7, 94.8, 88.4. APCI MS m/z [M + H]+: 298.1. HRMS (m/z): calculated for C_13_H_7_F_4_N_3_O: 297.0525: found: 297.0515.

^
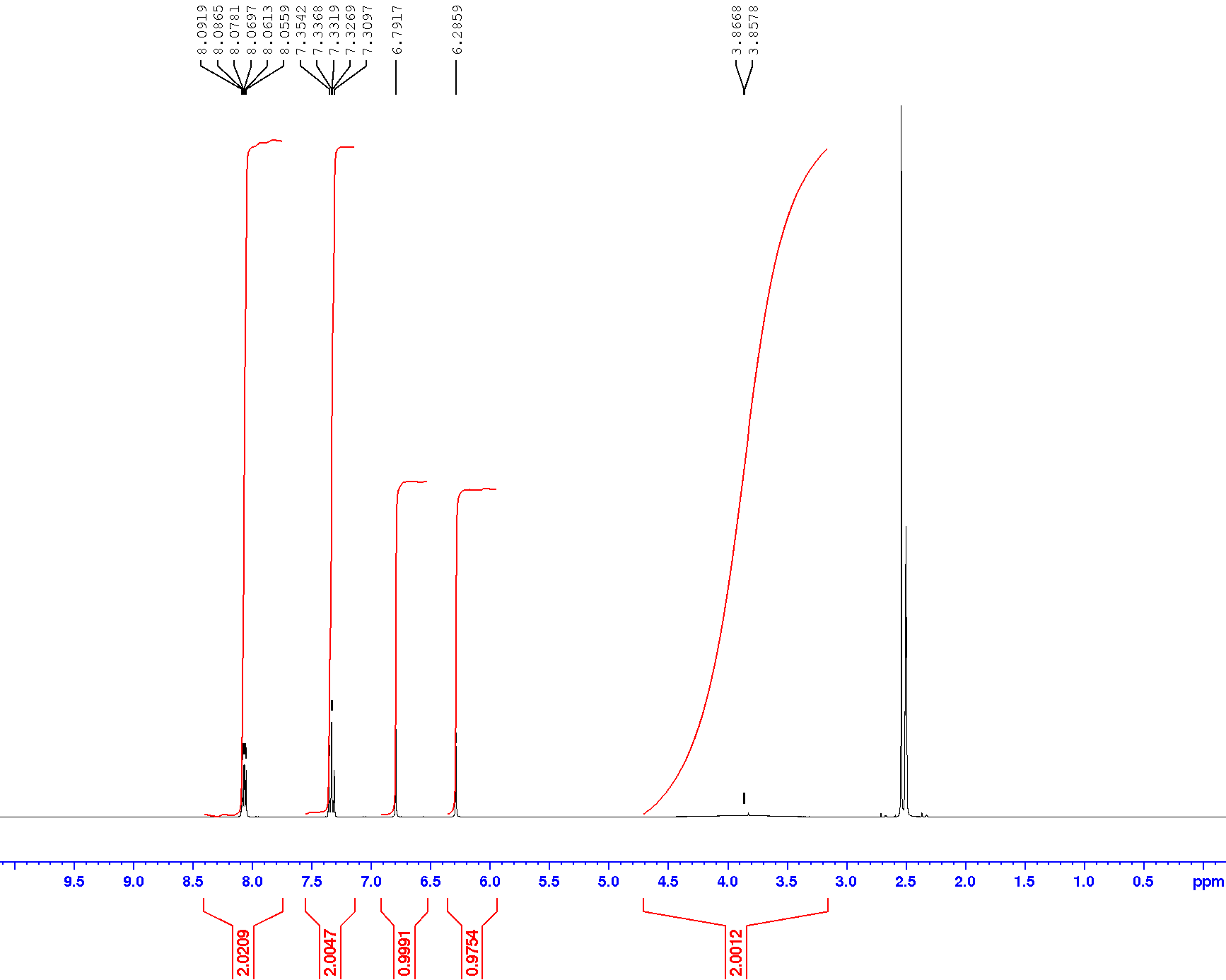
^

**Figure 10.** ^1^H NMR spectrum of **10**

**Figure 11.** ^13^C NMR spectrum of **10**

**7-Chloro-2-(4-fluorophenyl)-5-(trifluoromethyl)pyrazolo[1,5-α]pyrimidine 18**

2-(4-Fluorophenyl)-5-(trifluoromethyl)pyrazolo[1,5-α]pyrimidin-7(4*H*)-one **10** (0.70 g, 2.36 mmol) was added phosphorous oxychloride (5 mL) and the reaction was heated at 110 °C in a sealed tube for 12 h. Excess phosphorous oxychloride was removed by in vacuo and the mixture was partitioned between EtOAc and water, followed by washing with sat. NaHCO_3_ solution. The organic fractions were collected, dried and evaporated to give to give **18** (0.68 g, 92%) as a colourless solid which was used crude for the next step. APCI MS m/z [M + H]+: 316.1. HRMS (m/z): calculated for C_13_H_6_ClF_4_N_3_: 315.0186: found: 315.0196.

***N*-(6-(2-(Dimethylamino)ethoxy)-5-fluoropyridin-3-yl)-2-(4-fluorophenyl)-5-(trifluoromethyl)pyrazolo[1,5-α]pyrimidin-7-amine 2**

Chloride **18** (0.164 g, 0.521 mmol) in toluene (3 mL) was added 6-(2-(dimethylamino)ethoxy)-5-fluoropyridin-3-amine (0.125 g, 0.625 mmol) and flushed with nitrogen for 5 min. XPhos (0.050 g, 0.104 mmol) and Pd_2_dba_3_ (0.048 g, 0.052 mmol) were added to the reaction mixture and heated to 100 °C for 10 min. After 10 min, NaOtBu (0.100 g, 1.04 mmol) was added and the reaction was heated at 100 °C for further 2.5 h. Reaction mixture was concentrated under reduced pressure and purified by flash column chromatography with silica using EtOAc:MeOH (90:10) to give **2** (0.036 g, 25%). HPLC purity 95.5%. MP= 170-172 °C. ^1^H NMR (DMSO, 400 MHz) δ 10.41 (s, 1H), 8.22-8.18 (m, 2H), 8.17 (d, J = 2.2 Hz, 1H), 7.97 (dd, J = 11.1, 2.2 Hz, 1H), 7.42-7.37 (m, 2H), 6.36 (s, 1H), 4.50 (t, J = 5.8 Hz, 2H), 2.71 (t, J = 5.8 Hz, 2H), 2.27 (s, 6H). ^13^C NMR (DMSO-d6, 101 MHz) δ 164.1, 161.6, 154.7, 150.9, 150.9, 149.0, 147.4, 147.1, 146.5, 146.2, 144.8, 139.0, 139.0, 128.8, 128.7, 128.6, 128.6, 127.4, 122.8, 122.6, 122.5, 119.8, 115.9, 115.7, 93.6, 82.8, 64.3, 57.4, 45.4. APCI MS m/z [M + H]+: 479.2. HRMS (m/z): calculated for C_22_H_19_F_5_N_6_O: 478.1541: found: 478.1546.


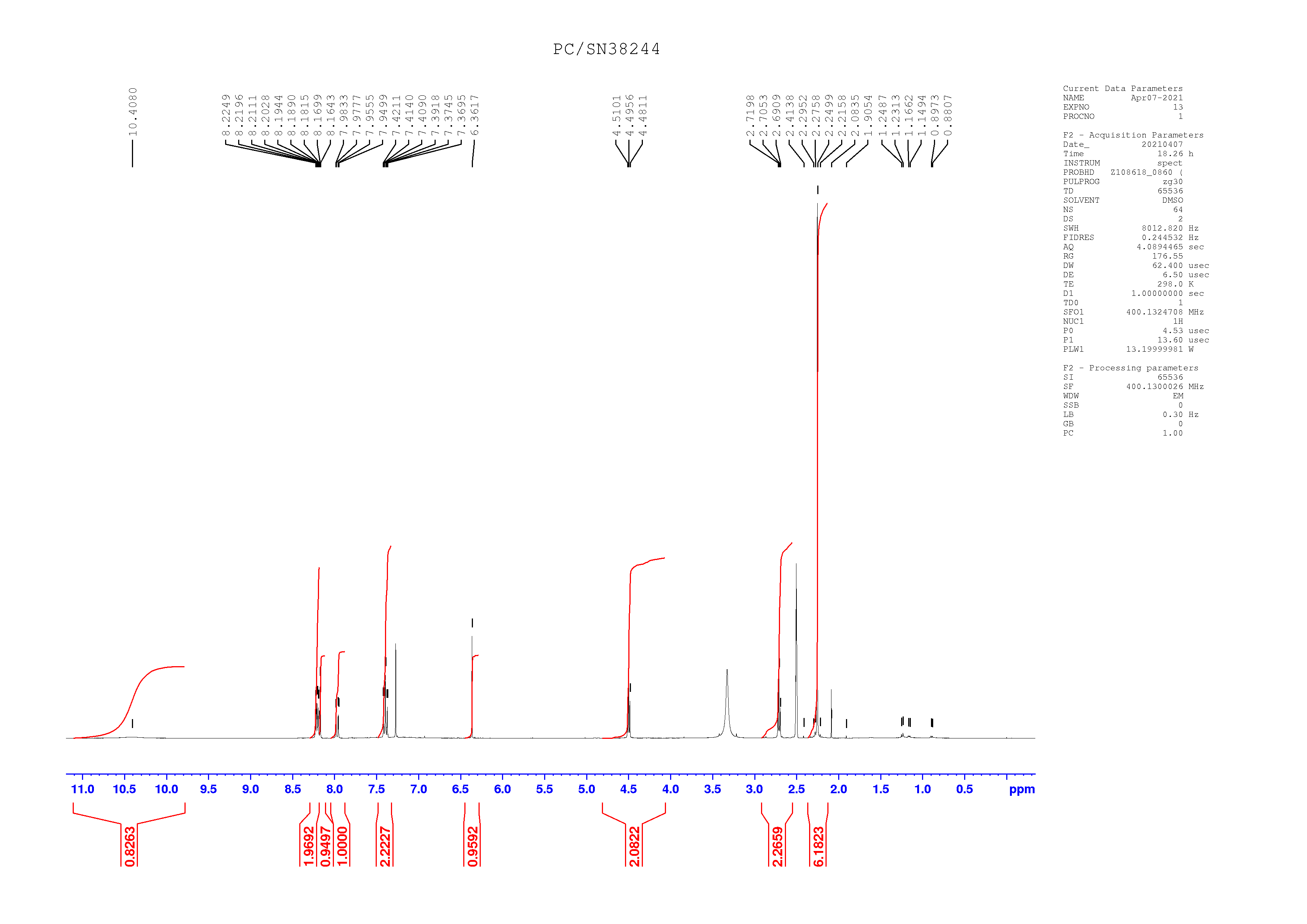


**Figure 12.** ^1^H NMR spectrum of **2**

**Figure 13.** ^13^C NMR spectrum of **2**

**References**

1. Kelada, M.; Walsh, J. M. D.; Devine, R. W.; McArdle, P.; Stephens, J. C., Synthesis of pyrazolopyrimidinones using a "one-pot" approach under microwave irradiation. *Beilstein J. Org. Chem.* **2018,** *14*, 1222-1228.

2. Tantry, S. J.; Shinde, V.; Balakrishnan, G.; Markad, S. D.; Gupta, A. K.; Bhat, J.; Narayan, A.; Raichurkar, A.; Jena, L. K.; Sharma, S.; Kumar, N.; Nanduri, R.; Bharath, S.; Reddy, J.; Panduga, V.; Prabhakar, K. R.; Kandaswamy, K.; Kaur, P.; Dinesh, N.; Guptha, S.; Saralaya, R.; Panda, M.; Rudrapatna, S.; Mallya, M.; Rubin, H.; Yano, T.; Mdluili, K.; Cooper, C. B.; Balasubramanian, V.; Sambandamurthy, V. K.; Ramachandran, V.; Shandil, R.; Kavanagh, S.; Narayanan, S.; Iyer, P.; Mukherjee, K.; Hosagrahara, V. P.; Solapure, S.; Hameed P, S.; Ravishankar, S., Scaffold morphing leading to evolution of 2,4-diaminoquinolines and aminopyrazolopyrimidines as inhibitors of the ATP synthesis pathway. *Med. Chem. Comm.* **2016,** *7* (5), 1022-1032.

3. Confirmation of condensation at the 4-position to yield pyrazolo[3,4-β]pyridinones such as **11**, is established by the presence of a distinctive NH proton at the δ 12-14 ppm region in the ^1^H NMR spectrum, whereas condensations at the 1-position to yield pyrazolo[1,5-α]pyrimidinone such as **6** or **10** do not have this NH peak.
